# Development and Validation of a Training Curriculum for Peripherally Inserted Central Catheter (PICC) Use Among Sudanese Health Professionals

**DOI:** 10.7759/cureus.93113

**Published:** 2025-09-24

**Authors:** Ragda Abdallah, Najla Mohammed, Mahil Abdalla, Ruaa Tagelsir Mustafa Abdelsalam, Yusra Ahmed Mohamedzein Adam, Mohamed Elsheikh, Rehab Musa, Fatima Ahmed, Lama Mohamed, Hanan Morsy

**Affiliations:** 1 Department of Pediatrics, Hull University Teaching Hospitals, Hull, GBR; 2 Department of Vascular Surgery, Norfolk and Norwich University Hospital Foundation Trust, Norwich, GBR; 3 Department of Emergency Medicine, Hull University Teaching Hospitals, Hull, GBR; 4 Department of Anaesthesiology and Intensive Care, Saudi Commission for Health Specialties (SCFHS), Riyadh, SAU; 5 Department of Physiology, Neuroscience, and Behavioral Sciences, St. George's University, St. George, GRD; 6 Department of Physiology, St. George’s University, St. George, GRD; 7 Department of Medicine, University of Khartoum, Khartoum, SDN; 8 Department of Obstetrics and Gynaecology, University Hospital Waterford, Waterford, IRL; 9 Department of Internal Medicine, University of Medical Sciences and Technology, Khartoum, SDN; 10 International Education Management (INEMA), Helwan University, Ludwigsburg University of Education, Helwan, EGY

**Keywords:** course curriculum, curriculum development, medical education, peripherally inserted central catheter (picc), : simulation-based training, sudanese health professionals, training, ultrasound-guided, vascular access device

## Abstract

Background: In Sudan, vascular access options are limited to short peripheral cannulas and untunneled central lines. Patients requiring peripherally inserted central catheters (PICCs) must often travel abroad, leading to unsafe practices and higher complication risks. Introducing PICCs requires a structured training program for local health professionals.

Objective: To design and validate a PICC training curriculum tailored for Sudanese healthcare providers.

Methodology: A cross-sectional study was conducted to develop a training curriculum. Curriculum design followed Kern’s six-step model, supplemented by Caffarella’s adult learning framework, and was benchmarked against international case studies. Problem identification and general needs assessment were undertaken through semi-structured interviews, while a targeted needs assessment was conducted using questionnaires to evaluate knowledge, skills, and training requirements among health professionals. Validation was carried out through expert review, using structured questionnaires and one-to-one interviews.

Results: Fifty-one health professionals participated in the needs assessment. Knowledge and skills were generally poor, with significant associations between knowledge deficits and fear of insertion (*P *= 0.007). Fear levels were higher among females (*P *< 0.05), varied by occupation (*P *= 0.002), and were influenced by years of experience (*P *< 0.05). Participants strongly preferred blended learning, with simulation and supervised practice rated highest. Seven field experts validated the curriculum and recommended shortening the timetable, enhancing infection prevention modules, and extending supervised practice requirements. The final curriculum comprised three days of lectures and clinical stations, followed by a six-month logbook for supervised insertions.

Conclusions: This study developed and validated Sudan’s first PICC training curriculum. The program combines theoretical instruction, simulation, and supervised practice, with contextual adaptations for feasibility. Implementation could improve patient safety, reduce complications, and strengthen national capacity in vascular access care.

## Introduction

Central venous catheterization is a common clinical procedure. It is commonly required for monitoring central venous pressure or for administering fluids, medications, chemotherapy, and parenteral nutrition over a prolonged period. Peripherally inserted central catheters (PICCs) are widely used and have partly replaced centrally inserted lines in many clinical settings [1].

Patients considered most suitable for PICC insertion include those requiring long-term vascular access, those receiving medications incompatible with peripheral intravenous routes, patients with an intended course of intravenous antibiotics longer than one week, individuals with difficult intravenous access (DIVA), and neonates admitted to the NICU. The main types of central venous catheterization include the PICC and the subcutaneous (implanted) port. Common veins used for central venous catheterization are the internal jugular, common femoral, and subclavian veins [2]. PICCs are valued for their reduced risk of procedural complications and catheter-related bloodstream infections [3].

Training for PICC insertion should be comprehensive, including didactic lectures, simulation-based skills training, and supervised clinical practice [4]. However, based on the researcher’s experience in Sudan, no formal training in PICC insertion is available, nor is it taught at the undergraduate level for medical professionals. This lack of structured knowledge has delayed the introduction and widespread use of PICCs in Sudan. Nevertheless, they are expected to be introduced into the Sudanese medical supply market in the near future.

International examples highlight the value of structured training. The MD Anderson Cancer Center has developed a multidisciplinary vascular access algorithm supported by standardized checklists, while Johns Hopkins Hospital requires competency-based training and supervised PICC insertions before certification [5,6]. These cases illustrate how structured curricula reduce complications and ensure safe practice.

Curriculum design for this study was guided by Kern’s six-step model, a widely applied framework in medical education that emphasizes problem identification, targeted needs assessment, setting goals and objectives, educational strategies, implementation, and evaluation with feedback [7]. This structured approach provided the foundation for developing a PICC training program tailored to the Sudanese healthcare context.

This research, therefore, aims to design a curriculum framework to train health professionals in Sudan on the insertion, care, and maintenance of PICCs, with the ultimate goal of improving patient care and broadening access to modern vascular access techniques.

## Materials and methods

Study design

This was a cross-sectional study aimed at developing a structured curriculum for training Sudanese health professionals in the insertion and care of PICCs. The proposed curriculum is intended for submission to the Sudan Medical Specialization Board and the Ministry of Higher Education for consideration and adoption.

Research tools

Problem identification and general needs assessment were conducted through semi-structured interviews with health professionals to evaluate their baseline knowledge of PICCs (Appendix A). A targeted needs assessment questionnaire was then distributed to identify training gaps and challenges (Appendix B). In addition, an expert validation questionnaire was used to assess the relevance and feasibility of the proposed program (Appendix C). A final one-on-one telephone meeting was conducted to obtain feedback on the PICC curriculum modules and topics. Finally, SPSS software, version 26 (IBM Corp., Armonk, NY), was employed for data entry, management, and statistical analysis.

Research procedure

Literature Review and Case Studies

A comprehensive review was conducted to examine international experiences with PICC use and training programs. As part of this review, two case studies were used as benchmarks.

MD Anderson Cancer Center: It developed a multidisciplinary vascular access algorithm supported by standardized insertion checklists, emphasizing patient assessment and infection prevention [5].

Johns Hopkins Hospital: It issued a vascular access policy requiring competency-based training and multiple supervised PICC insertions before certification, with clear supervisor qualifications [6].

Lessons from these models were adapted to the Sudanese context, ensuring alignment with international best practices while remaining feasible in the local context.

Curriculum Development

Curriculum development was primarily guided by Kern’s six-step model of medical education [7], supplemented by elements from Caffarella and Daffron’s Planning Programs for Adult Learners framework to address adult learning considerations in resource-limited contexts [8]. The Kern’s six steps are as follows:

Problem identification and general needs assessment: Semi-structured interviews were conducted with 20 registrars (ICU, pediatrics, and general practice). Variables collected included occupation, gender, and baseline knowledge regarding PICCs (awareness, importance, insertion, and care).

Targeted needs assessment: A validated online questionnaire was distributed to 51 health professionals (residents, nurses, general practitioners). Domains included demographics, knowledge of indications and complications, technical skills, intellectual factors (fears, motivation, attitudes), and preferred learning methods (lectures, mannequins, cadavers). Consent was obtained from all participants.

Goals and objectives: Learning outcomes were structured across cognitive (knowledge of anatomy, indications, and complications), psychomotor (PICC insertion, maintenance, and ultrasound guidance), and affective domains (confidence, patient safety, and adherence to standards). SMART criteria were applied to ensure measurable outcomes.

Educational strategies: A blended model was chosen: face-to-face and online lectures, simulation-based practice with mannequins, role-play of clinical scenarios, and supervised insertions.

Implementation: Implementation planning included preparatory steps such as identifying required resources (faculty, time, and facilities), establishing administrative support, outlining communication strategies, and anticipating potential barriers, including limited funding and institutional resistance. A pilot phase was proposed as a prerequisite before any full-scale implementation.

Evaluation and feedback: The draft curriculum (Appendix D) was evaluated by experts. Seven field experts from intensive care, pediatrics, radiology, anatomy, and medical education reviewed the draft curriculum to validate its content and structure. In addition, student performance assessment methods were built into the curriculum design to evaluate both theoretical knowledge and practical skills. A framework for post-implementation evaluation was also planned, including pre- and post-course surveys and participant feedback forms, to guide future refinements.

Contextual Adaptation

The draft curriculum was adapted in advance to reflect the economic, cultural, and clinical realities of the Sudanese healthcare system. Key considerations included limited financial resources, the absence of routine PICC availability, and workforce constraints.

Expert Validation

A cross-sectional validation study was carried out with seven experts in fields directly related to PICC use and insertion. The panel included specialists in intensive care, pediatrics, radiology, anatomy, and medical education. Each expert was invited to review the proposed curriculum and provide structured feedback on its content and feasibility.

A questionnaire, distributed via Google Forms, was developed to evaluate six core components of the curriculum: timetable, assessment and selection, insertion, post-insertion, implementation, and evaluation. Within each component, lecture topics and clinical stations were presented for review, and experts were asked to indicate whether each item was suitable, suitable to some extent, or not suitable, with proposed modifications.

Responses were analyzed using the Lawshe Content Validity Ratio (CVR) method, which quantifies expert agreement on items [9]. According to this approach, items with CVR values ≥0.85 were considered valid, while those below this threshold were classified as failed and required revision or removal. Following completion of the survey, one-to-one telephone interviews were conducted to obtain more detailed feedback and to reach consensus on the final refinements of the curriculum.

**Figure 1:**
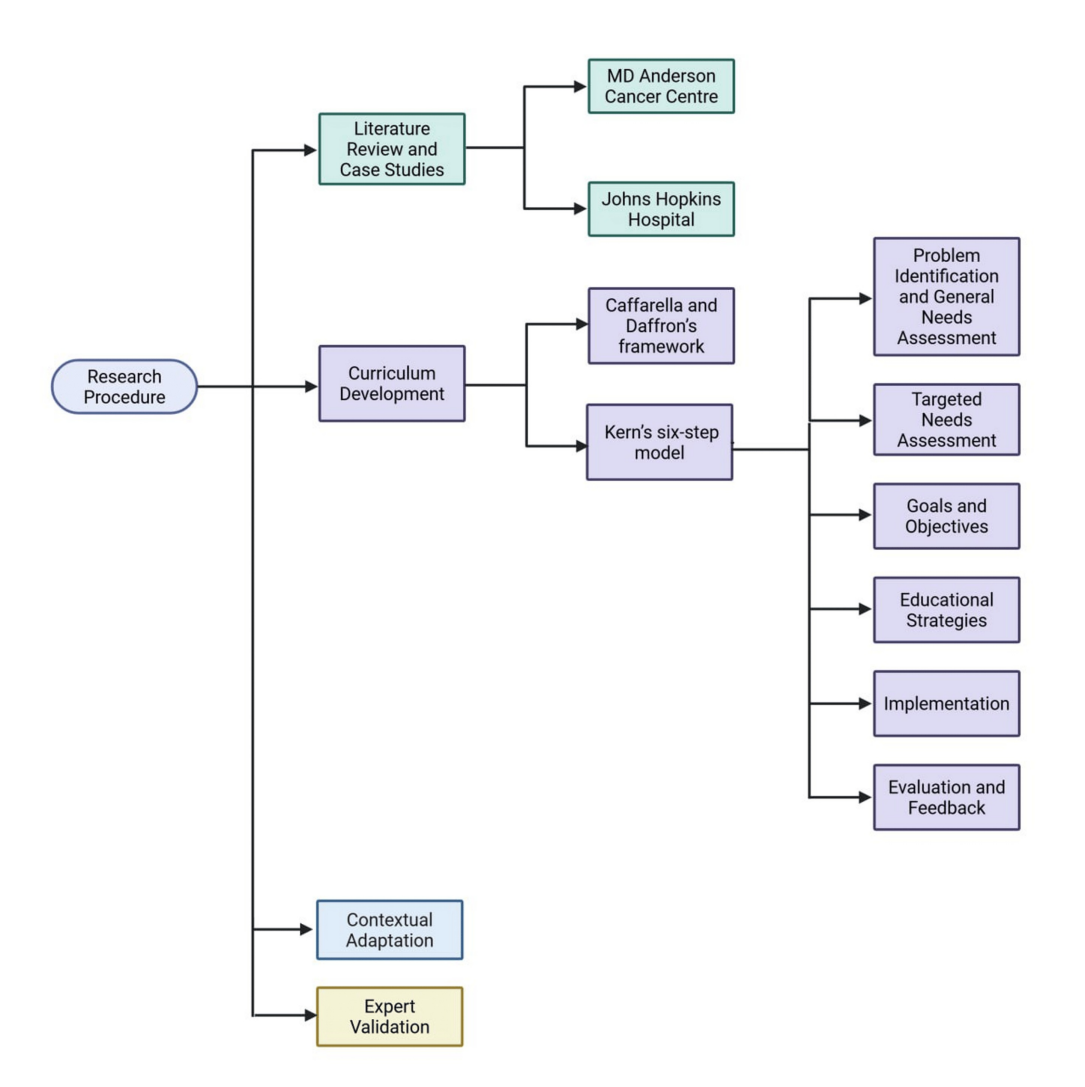
Curriculum development process for PICC training in Sudan. Schematic representation of the stepwise methodology used to design and validate the PICC training curriculum. The process included (1) literature review and international case studies (MD Anderson Cancer Center [5], Johns Hopkins Hospital [6]); (2) curriculum development guided by Kern’s six-step model [7] and supplemented by Caffarella and Daffron’s adult learning framework [8]; (3) contextual adaptation to Sudanese healthcare realities; and (4) expert validation through questionnaires and follow-up interviews. Image credit: All authors. PICC, peripherally inserted central catheter

Figure 1 illustrates the curriculum development process.

## Results

Literature review and case studies

The literature review confirmed a lack of PICC training programs in Sudan and limited awareness among healthcare professionals. International case studies (MD Anderson and Johns Hopkins) highlighted the importance of standardized protocols, competency-based training, and supervised practice, which were incorporated into the proposed curriculum design.

Curriculum development (Kern’s six steps applied)

Problem Identification and General Needs Assessment

Semi-structured interviews with 20 registrars (ICU, pediatrics, general practice) revealed that only four participants had ever heard of PICCs and none had insertion experience. Most were unaware of device care, complication management, or clinical indications.

Targeted Needs Assessment

A total of 51 health professionals (residents, nurses, and general practitioners) completed the online targeted needs assessment questionnaire. Participant characteristics are summarized in Table 1.

**Table 1:** Participant demographics (n = 51). Characteristics of the 51 health professionals who completed the targeted needs assessment questionnaire. Gender, occupation, and years of experience were explored in relation to knowledge and fear of PICC insertion. PICC, peripherally inserted central catheter

Variable	Category	*n* (%)	Key findings
Gender	Male	27 (53%)	Females showed significantly lower knowledge and higher fear (*P* < 0.05).
	Female	24 (47%)	
Occupation	Residents	20 (39%)	Occupation was significantly associated with fear of insertion (*P* = 0.002).
	Nurses	15 (29%)	
	General practitioners	10 (20%)	
	Other	6 (12%)	
Years of experience	<5 years	28 (55%)	No significant link with knowledge, but significant association with fear (*P* < 0.05).
	≥5 years	23 (45%)	

Most participants had poor or weak knowledge of PICCs. Many could not identify suitable veins, indications, or patient groups. Knowledge deficits were significantly associated with higher fear of insertion (*P* = 0.007). Years of experience and occupation did not affect knowledge levels. Gender differences were significant, with female participants showing lower knowledge (*P* < 0.05). Associations between knowledge and fear are shown in Table 2.

**Table 2:** Knowledge vs. fear associations. Chi-square analysis demonstrated that lower knowledge was significantly associated with higher fear of PICC insertion (*P *= 0.007). Gender differences were significant, whereas years of experience and occupation showed no significant effect on knowledge.

Variable comparison	Association result	*P*-value
Knowledge vs. fear	Poor knowledge = higher fear	0.007
Knowledge vs. years of experience	No significant relationship	0.915
Knowledge vs. occupation	No significant relationship	0.925
Knowledge vs. gender	Gender difference significant	<0.05

Self-reported skills in insertion, maintenance, and complication management were very low. Lack of skills correlated strongly with increased fear of insertion (*P* < 0.05). These findings are presented in Table 3.

**Table 3:** Skills and attitudes toward PICCs. Most participants reported low skills in PICC insertion, maintenance, and complication management. High levels of fear were reported and were significantly influenced by gender, occupation, and years of experience. PICC, peripherally inserted central catheter

Variable	Category	*n* (%)	Key findings
Insertion skills	Adequate	5 (10%)	Most participants had no prior insertion experience.
	Inadequate	46 (90%)	
Skills in PICC care/maintenance	Adequate	8 (16%)	Overall competence in care and maintenance was poor.
	Inadequate	43 (84%)	
Skills in complication management	Adequate	6 (12%)	Limited knowledge of managing complications.
	Inadequate	45 (88%)	
Attitudes/Fear of insertion	Reported fear	35 (69%)	Fear was associated with gender, occupation, and years of experience (P < 0.05).
	No fear	16 (31%)	

Many participants reported fear of insertion, citing technical difficulty and risk of complications. Fear levels differed significantly by gender (*P* = 0.05), occupation (*P* = 0.002), and years of experience (*P* < 0.05).

Regarding educational preferences, respondents favored blended learning, including lectures, simulation on mannequins, and supervised insertions. Preferences are detailed in Table 4.

**Table 4:** Educational preferences for PICC training (n = 51). Participants indicated a strong preference for blended learning methods, with emphasis on lectures for theoretical knowledge, simulation-based practice using mannequins, supervised clinical insertions, and role-play of clinical scenarios. PICC, peripherally inserted central catheter

Preferred training method	n (%)	Response trend
Lectures (face-to-face/online)	40 (78%)	Favored for the theoretical foundation
Simulation (mannequins)	47 (92%)	Strongly preferred before patient contact
Supervised clinical insertions	45 (88%)	Viewed as essential for competence
Role-play scenarios	30 (59%)	Supported for practicing complication management

*Goals and Objectives* Based on needs assessment results, specific objectives were formulated to address knowledge gaps (e.g., anatomy, indications, complications), develop practical skills (insertion, maintenance, ultrasound use), and improve confidence and patient safety attitudes.

*Educational Strategies* Participants endorsed the use of simulation and supervised practice before clinical insertion. Simulation-based training with mannequins, supported by lectures and role-play scenarios, was prioritized in the proposed design.

*Implementation* A final validated three-day curriculum was developed following the evaluation and expert validation phases. The final curriculum was not piloted within the scope of this study; however, the complete timetable, course structure, assessment framework, and certification criteria are provided in Appendices E-G to facilitate replication and future implementation**.**

Evaluation and Feedback

Expert validation was successfully completed. Feedback from seven experts informed refinements to the timetable, module sequencing, infection prevention content, and supervised practice requirements, which were incorporated into the final version of the curriculum. While student performance assessments and program evaluation mechanisms (including pre- and post-course surveys and participant feedback forms) were designed, these were not applied within the scope of this study and remain planned for future implementation.

Contextual adaptation

Adaptations included limiting curriculum duration to three days, reducing reliance on costly resources, and emphasizing simulation-based teaching to address limited patient access. These modifications ensured cultural and economic feasibility in Sudan.

Expert validation

A total of seven field experts reviewed the draft curriculum. The validation questionnaire, analyzed using the Lawshe CVR, identified several items with CVR values below the cut-off point of 0.85. These included the timetable (-0.333, Failed), Lecture I: Vascular Anatomy and Physiology (0.667, Failed), Lecture III: PICC Insertion Preparation (0.667, Failed), Station 5: Complications (0.667, Failed), Station 7: Hub Disinfection, Management, and Flushing (0.667, Failed), and Day 5: Assessments (0.667, Failed).

During follow-up telephone interviews, experts recommended modifications to these areas rather than their exclusion. Specifically, the timetable was shortened to three days, lectures on anatomy and insertion preparation were reorganized, infection prevention content (including hub disinfection and flushing) was expanded, and the logbook requirement was extended to six months with at least five supervised insertions.

All other items achieved CVR values of 1.0 (Passed) and were retained without major change. Importantly, the experts endorsed the overall curriculum structure and blended teaching approach as appropriate and aligned with international standards.

The CVR results and final expert-driven modifications are presented in Table 5.

**Table 5:** Expert validation feedback and final modifications (n = 7 experts). Items with CVR < 0.85 were considered *Failed*. *Passed* items were retained without major modification. For failed items, expert interviews guided revisions (shown in *Final Modification*). CVR, Content Validity Ratio (Lawshe method)

Curriculum element	CVR value	Lawshe result	Final modification
Timetable	-0.333	Failed	Course duration reduced to 3 days to improve feasibility and participant attendance
Lecture I: Vascular Anatomy and Physiology	0.667	Failed	Content reorganized and clarified; retained after expert consensus
Lecture II: Vascular Access Devices	1.0	Passed	Retained with no major changes.
Lecture III: PICC Insertion Preparation	0.667	Failed	Expanded and reorganized within Day 1 sessions
Station 1: Ultrasound Guided Insertion	1.0	Passed	Retained with no major changes
Station 2: Tip Position	1.0	Passed	Retained with no major changes
Station 3: Securement and Dressing	1.0	Passed	Retained with no major changes
Station 4: Infection Prevention	1.0	Passed	Retained with no major changes
Station 5: Complications	0.667	Failed	Expanded with more emphasis on complication prevention and management
Station 6: Catheter Function and Device Necessity	1.0	Passed	Retained with no major changes
Station 7: Hub Disinfection, Management, and Flushing	0.667	Failed	Expanded with practical infection-prevention skills
Day 4: Implementation (Role Play)	1.0	Passed	Retained with no major changes
Day 5: Assessment (Theory, Practical, Logbook)	0.667	Failed	Logbook requirement extended to 6 months with ≥5 supervised insertions

Curriculum output

The final product of this study was a structured curriculum designed to train Sudanese health professionals in the safe insertion and management of PICCs. The full curriculum outline is provided in Appendices E-F, and the assessment and certification criteria are detailed in Appendix G.

*Course Description and Purpose* The curriculum equips participants with the theoretical knowledge, practical skills, and professional attitudes required for safe and effective PICC insertion. Its primary aim is to improve clinical practice in Sudan by increasing insertion success rates and reducing complications and costly referrals abroad.

*Course Design and Structure* The training integrates didactic modules with skills-based practice and supervised clinical exposure. Content is organized stepwise, progressing from device selection and patient assessment to insertion, maintenance, and management of complications.

Duration and Timetable

The curriculum is delivered over three days. Day 1 covers theoretical modules on anatomy, vascular devices, and insertion preparation, followed by skills labs on ultrasound guidance, tip positioning, securement, and infection prevention. Day 2 includes clinical stations on post-insertion care, complication management, catheter function, hub disinfection, and role-play scenarios. Day 3 focuses on supervised patient demonstrations, small-group practice, and summative assessments.

In total, the course comprises 7.5 hours on Day 1, 7.5 hours on Day 2, and 8 hours on Day 3, including breaks. Following course completion, participants are required to maintain a six-month logbook documenting at least five supervised patient insertions. A summary of the timetable is shown in Appendix E, with full details presented in Appendix F.

*Target Group* The course is intended for acute and senior residents in vascular surgery, intensive care, anesthesia, pediatrics, medicine (including interventional radiology), as well as nurses and advanced medical students. To optimize learning, group size is limited to 15 participants, subdivided into groups of five for clinical stations.

Learning Outcomes

Knowledge: Anatomy, PICC indications, device selection, insertion preparation, complication management, and infection prevention. Skills: Ultrasound-guided insertion, tip positioning, securement, dressing, flushing, and complication management. Attitudes/professional development: Confidence in insertion, adherence to sterile technique, and commitment to safe vascular access practice.

Assessment and Certification

Certification is awarded to participants who meet the following criteria: attend at least 75% of the course; achieve a minimum score of 70% in the theoretical exam; complete five mannequin insertions (with up to two reattempts if required); and successfully perform five supervised patient insertions within six months. All practical sessions, including mannequin practice and patient insertions, were conducted under direct hands-on supervision by certified instructors. Skills acquisition was assessed using structured checklists during simulation (Objective Structured Clinical Examination (OSCE)-based assessments) and validated logbooks for patient insertions, ensuring standardization and feedback-driven improvement. Assessment components, time allocations, and passing requirements are detailed in Appendix G.

## Discussion

This study developed and validated a structured curriculum for training Sudanese health professionals in the insertion and care of PICCs. The needs assessment revealed limited knowledge, skills, and confidence among participants, despite widespread recognition of the importance of PICCs in clinical practice. Fear of insertion was common and significantly associated with gender, occupation, and years of experience. Preferences for training strongly favored blended approaches, including lectures, simulation-based practice, and supervised insertions. Expert validation confirmed the feasibility of the curriculum, while recommending refinements in timetable, infection prevention, and supervised practice requirements.

These findings are consistent with international reports that highlight deficits in vascular access knowledge and the impact of structured training programs. Previous studies have demonstrated that inadequate training contributes to higher complication rates and reduced clinician confidence, whereas competency-based education significantly improves safety and outcomes [1,4,7,10]. Similarly, a study from Egypt found that targeted educational programs significantly improved nurses’ knowledge and practice regarding vascular access [11].

The strong preference for simulation-based training in this study mirrors global trends in medical education. Simulation has been shown to enhance procedural competence and reduce anxiety before patient-based practice [12]. Studies comparing simulation-based training with traditional methods in central line placement also demonstrated improved proficiency and reduced complication rates among trainees [13].

The application of Kern’s six-step model provided a systematic framework for curriculum design, ensuring that the process was evidence-based, learner-focused, and adaptable [7]. Supplementing this with Caffarella and Daffron’s adult learning framework helped address learner motivation and contextual challenges specific to low-resource settings [8]. International examples further guided development: the MD Anderson vascular access algorithm and Johns Hopkins Hospital vascular access policy both stress standardization, supervised practice, and competency-based certification [5,6]. Incorporating these lessons strengthened the Sudanese curriculum by aligning it with international best practices.

Contextual adaptation was crucial, given Sudan’s unique healthcare challenges. Limited availability of PICCs, scarcity of trained personnel, and financial barriers to prolonged training necessitated a concise but effective course design. By limiting the course to three days, emphasizing simulation and supervised practice, and extending the logbook requirement, the final curriculum balances feasibility with quality assurance. Expert consensus confirmed that these adaptations preserved international standards while addressing local realities.

This study has several strengths. It applied a validated medical education framework, integrated principles of adult learning, and involved both physicians and nurses in the needs assessment. In addition, expert validation ensured that the curriculum was feasible and clinically relevant. However, some limitations must be acknowledged. The sample size of the needs assessment was modest, and participants were drawn from selected institutions, potentially limiting generalizability. Furthermore, the curriculum has not yet been piloted, and its effectiveness in improving clinical outcomes and reducing complications remains to be evaluated. Additionally, as the study relied on self-reported skills and attitudes, there is potential for response bias. Furthermore, while the curriculum was adapted to Sudan’s context, cultural and institutional barriers to adoption, such as resistance to new training approaches, financial constraints, and administrative challenges, were not fully examined in this study and may influence future implementation.

The validated curriculum represents the first structured PICC training program developed for Sudan. By combining theoretical instruction, simulation-based training, and supervised clinical practice, it offers a feasible pathway to improving vascular access safety and reducing complications. Its structured design, adapted from Kern’s model and supported by international case studies, ensures alignment with global best practices while addressing Sudan’s local realities. If implemented, this program could reduce unnecessary overseas referrals, strengthen healthcare workforce capacity, and serve as a model for future specialty training curricula in the region.

Future directions

Piloting of the curriculum is recommended to evaluate its effectiveness in improving procedural competence, reducing complication rates, and supporting integration into postgraduate and continuing professional development programs.

## Conclusions

This study developed and validated the first structured curriculum for PICC training in Sudan. The needs assessment revealed significant gaps in knowledge, skills, and confidence, while expert review ensured the curriculum’s feasibility, relevance, and alignment with international standards. The program combines theoretical instruction, simulation-based practice, and supervised insertions, with contextual adaptations to address local healthcare realities. Implementation of this curriculum has the potential to improve patient safety, reduce complications, and build national capacity in vascular access practice.

## Appendices

Appendix A 

**Table 6:** Semi-Structured Interview Questions for Problem Identification and General Needs Assessment

Domain	Interview Questions
Demographics	Occupation
	Gender
Knowledge	Do you know what a PICC is?
	Do you know the importance of PICC?
	Do you know how to insert a PICC?
	Do you know how to care for a PICC?

Appendix B

**Table 7:** Peripherally Inserted Central Catheters (PICC) Use Targeted Needs Assessment Questionnaire This questionnaire aims to assess the Knowledge, Attitude, and Practices of the Sudanese Health professionals regarding Peripherally Inserted Central Catheters (PICC) use.

Section	Question	Response options
Consent	By filling this questionnaire you agree to participate in this research project. Note: Any information given in this questionnaire will be confidential and will be used for research purposes only.	Agree / Disagree
Demographics	Occupation	Nurses/General practitioners/Residents/ other
	Years of experience	Less than 5 years/ More or equal to 5 years
	Gender	Male / Female
Knowledge	Do you know what is a PICC?	Yes / No
	How do you describe your knowledge about PICCs?	Weak / Good / Excellent
	How do you describe your knowledge about the types of patients who need PICCs?	Weak / Good / Excellent
	How do you describe your knowledge about the types of veins appropriate for PICC insertion?	Weak / Good / Excellent
	How do you describe your knowledge about caring for a PICC?	Weak / Good / Excellent
	How do you describe your knowledge about maintaining the device through daily assessment of the site, function, and dressing?	Weak / Good / Excellent
	How do you describe your knowledge about giving fluids through a PICC?	Weak / Good / Excellent
	How do you describe your knowledge about PICC complications?	Weak / Good / Excellent
	How do you describe your knowledge about managing PICC complications?	Weak / Good / Excellent
	How do you evaluate the necessity of a PICC (whether it is still needed or not)?	Weak / Good / Excellent
Attitudes	Are you convinced that the PICC is important in certain situations?	Yes / No / I do not know
	Do you have any fears regarding inserting a PICC?	Yes / No / I do not know
	What is the most worrying thing about inserting a PICC?	Open response
Preferred learning methods	Choose the mode of learning you prefer for the theoretical part of PICC insertion	Face-to-face lectures / Live online lectures / Recorded video lectures / online and face-to-face lectures/ only online lectures
	Choose how you prefer to learn the skills of PICC insertion	Manikins / Cadavers
	Choose how you prefer to practice complication management	Role play/ Case-based discussion /Lectures
	Choose your preferred method to assess competency	Successful completion of the course / Supervised clinical insertions
Practices	Do you think it is important to assess the side of the PICC?	Yes / No / I do not know
	Do you think it is important to assess the dressing of PICCs?	Yes / No / I do not know
Training needs	Do you want to know how to insert a PICC?	Yes / No / I do not know
	Do you think this training will increase your skills in inserting PICCs?	Yes / No / I do not know

Appendix C

**Table 8:** Experts Validation Questionnaire for the Peripherally Inserted Central Catheters (PICC) Use Draft Curriculum

Section	Item	Description	Expert Response Options	Modifications, additions, or comments
General	Consent	By filling out this questionnaire, you agree to participate in this research project. Note: Any information given in this questionnaire will be confidential and will be used for research purposes only.	Agree / Disagree	
Timetable	Time Table of the PICC Use Training	Day 1: Assessment & Selection Lecture I: Understanding Vascular Anatomy and Physiology: 45 mins Lecture II: Selecting a Vascular Access Device: 45 mins Lecture III: Preparing for PICC Insertion: 45 mins	Suitable / To some extent suitable / Not suitable	
		Day 2: Insertion Clinical Station 1: Step by Step Ultrasound Guided PICC Insertion – 45 mins Clinical Station 2: Tip Position – 45 mins Clinical Station 3: Right Securement, Dressing, and Management – 45 mins Clinical Station 4: Right Infection Prevention – 45 mins	Suitable / To some extent suitable / Not suitable	
		Day 3: Post insertion Clinical Station 5: Managing complications: 45 mins Clinical Station 6: Assessment for Catheter Function, and Device Necessity: 45 mins Clinical Station 7: Right Hub Disinfection, Management, and Flushing: 45 mins Practical: Skill lab practical implementation: 2 hours	Suitable / To some extent suitable / Not suitable	
		Day 4: Implementation Role Play 1: Case scenarios example 1: 30 mins Role Play 2: Case scenarios example 2: 30 mins Role Play 3: Case scenarios example 3: 30 mins Practical: Skill lab practical implementation: 2 hours	Suitable / To some extent suitable / Not suitable	
		Day 5: Assessments Assessment 1 Theoretical Assessment: 30 mins Assessment 2 Practical Assessment: 30 mins Assessment 3 Logbook Completion: 1 month	Suitable / To some extent suitable / Not suitable	
Day 1: Assessment & Selection	Lecture I. Vascular Anatomy and Physiology	Topic Description: Deliver a general understanding of the Anatomy and Physiology of the circulatory system. Topics covered: Introduction; Vessel Wall Structure; Vascular features; Large veins of the Upper Arm; Vessels for PICC Insertion; Vessels of the Thorax; Nerves of the Upper Arm; Physiology of the Venous System; Virchow’s Triad; Variations by Age Group (Neonatal, Infants, Toddler, Children, Adolescents); Key Takeaways	Suitable / To some extent suitable / Not suitable	
	Lecture II. Vascular Access Devices	Topic Description: Discusses the different types of vascular devices and how to choose the appropriate device. Topics covered: Introduction; Types of Vascular Access Devices; PICCs; Device-Specific Features (Multi-lumen catheters, size, dialysis/apheresis catheters); Infusate Features; Catheter Features; Length of Therapy; Device selection by patient assessment; Pediatric Patients; Key Takeaways	Suitable / To some extent suitable / Not suitable	
	Lecture III. PICC Insertion Preparation	Topic Description: Required steps to evaluate the patient, educate the patient, obtain informed consent, perform the maximal sterile barrier, and verify appropriateness. Topics covered: Introduction to Insertion; Informed Consent; Pre-Insertion Assessment; Patient and clinician Education; Positioning and Measuring Techniques; Insertion Environment; Local Anaesthetic; Maximal Sterile Barrier (M6B) Precautions; Number of Access Attempts; Seldinger Technique	Suitable / To some extent suitable / Not suitable	
Day 2: Insertion	Station 1. Ultrasound Guided PICC Insertion	Topic Description: Bedside use of ultrasound for PICC placement, vessel identification, and avoiding blind insertion. Topics covered: Introduction to Ultrasound; Getting Started; Choosing a Vein; Imaging Veins; Using the Probe; Image Optimisation; Modified Seldinger Insertion; Step by Step Explanation	Suitable / To some extent suitable / Not suitable	
	Station 2. Tip Position	Topic Description: Vitality of PICC insertion confirmation through Radiography and ECG. Topics covered: Tip positioning; Radiography; ECG guidance; Guidewire technique; Saline-filled lumen technique; Tip movement; Thrombosis; Conclusion	Suitable / To some extent suitable / Not suitable	
	Station 3. Securement and Dressing	Topic Description: Proper care, securement, and dressing to detect complications early. Topics covered: Overview; Purpose of Securement; Types (Gauze, Transparent, Adhesive, Tissue adhesive, Subcutaneous, Sutures, Add-on devices); Impact of inadequate securement; Dressing assessment and changes	Suitable / To some extent suitable / Not suitable	
	Station 4. Infection Prevention	Topic Description: Aseptic care to maintain PICC use. Topics covered: Hand hygiene; Sterile gown/gloves/drapes; Patient skin preparation; Skin decontamination; Ultrasound; Insertion checklists; ANTT technique	Suitable / To some extent suitable / Not suitable	
Day 3: Post insertion	Station 5. Complications	Topic Description: Complications during or after insertion and their management. Topics covered: Insertion-related; Post-insertion; Any time; Summary	Suitable / To some extent suitable / Not suitable	
	Station 6. Catheter Function and Device Necessity	Topic Description: When and how to safely remove a PICC; assess function and complications. Topics covered: Assessment frequency; Insertion site inspection; Tubing/giving set labelling; Occlusions (thrombotic/non-thrombotic); Patient safety; Extravasation/infiltration; PPE; Aseptic technique (Device necessity; Care bundles)	Suitable / To some extent suitable / Not suitable	
	Station 7. Hub Disinfection, Management, and Flushing	Topic Description: Hub disinfection, site assessment, flushing methods. Topics covered: Disinfectants; Methods; Changing connectors; Site assessment and documentation; Flushing rationale and principles; Pre-filled syringes; Positive pressure flushing; Blood reflux; Needle-free connectors; Catheter valves; Flushing volumes; Frequency; Heparin vs saline; Clearing occlusions	Suitable / To some extent suitable / Not suitable	
Day 4: Implementation	Implementation and Role Play	Role Play: 3 scenarios (full insertion, complication during insertion, post-insertion complication). Practical implementation of the whole PICC process (insertion, management, removal). Adaptable to group preferences.	Suitable / To some extent suitable / Not suitable	
Day 5 Assessments	Final Assessment	Assessment 1: Theoretical Assessment (30 mins) Assessment 2: Practical Assessment (30 mins) Assessment 3: Logbook Completion (1 month)	Suitable / To some extent suitable / Not suitable	

Appendix D 

**Table 9:** Draft Curriculum

Day / Module	Topic	Content (Topic Description + Topics to be covered)	Time	Teaching Method / Learning Outcomes
Day 1: Assessment & Selection	Lecture I: Vascular Anatomy and Physiology	Topic Description: This topic is planned to deliver a general understanding of the Anatomy and Physiology of the circulatory system. Topics to be covered: Introduction; Vessel Wall Structure; Vascular features; Large veins of the Upper Arm; Vessels for PICC Insertion; Vessels of the Thorax; Nerves of the Upper Arm; Physiology of the Venous System; Virchow’s Triad; Vascular Access-Related Anatomical, Physiological, and Developmental Variations by Age Group: Neonatal, Infants, Toddler, Preschool, Adolescents; Key Takeaways.	45 mins	Teaching Method: Lecture. Learning Outcomes: 1. Knowledge and Understanding: Identify vessel-wall structure, veins, and surrounding anatomy. 2. Intellectual Skills: Analyse venous anatomy and physiology in relation to PICC placement. 3. General and Transferable Skills: Characterise appropriate vein selection behaviour.
Day 1: Assessment & Selection	Lecture II: Vascular Access Devices	Topic Description: This topic discusses the different types of vascular devices and how to choose the appropriate device according to the patients. Topics to be covered: Introduction; Types of Vascular Access Devices; PICCs; Device-Specific Features (multi-lumen catheters, catheter size, dialysis/apheresis catheters); Infusate Features; Catheter Features; Length of Therapy; Device selection by patient assessment; Pediatric Patients; Key Takeaways.	45 mins	Teaching Method: Lecture. Learning Outcomes: 1. Knowledge and Understanding: Identify vascular device types, features, and selection criteria. 2. Intellectual Skills: Analyse factors influencing device choice, including pediatric variation. 3. General and Transferable Skills: Select suitable devices according to patient's condition.
Day 1: Assessment & Selection	Lecture III: PICC Insertion Preparation	Topic Description: This topic will discuss the required steps to evaluate the patient, educate the patient, obtain informed consent, perform the maximal sterile barrier, and verify appropriateness of the procedure. Topics to be covered: Introduction to Insertion; Informed Consent; Pre-Insertion Assessment; Patient and clinician Education; Positioning and Measuring Techniques; Insertion Environment; Local Anaesthetic; Maximal Sterile Barrier (M6B) Precautions; Number of Access Attempts; Seldinger Technique.	45 mins	Teaching Method: Lecture. Learning Outcomes: 1. Knowledge and Understanding: Identify pre-insertion assessments and device choice. 2. Intellectual Skills: Analyse assessments, equipment, and sterilisation. 3. General and Transferable Skills: Promote a sterile environment for PICC insertion.
Day 2: Insertion	Station 1: Ultrasound Guided PICC Insertion	Topic Description: This topic focuses on the bedside use of ultrasound for PICC placement, in vessel identification to help in visualisation, to avoid blind insertion. Topics to be covered: Introduction to Ultrasound; Getting Started; Choosing A Vein; Imaging Veins; Using the Probe; Image Optimisation; Modified Seldinger Insertion; Step by Step Explanation.	45 mins	Teaching Method: Clinical station (knowledge, skills demonstration, practice). Learning Outcomes: 1. Knowledge and Understanding: Outline ultrasound basics. 2. Intellectual Skills: Analyse step-by-step US-guided insertion. 3. General and Transferable Skills: Perform probe/needle handling, follow tip on ultrasound.
Day 2: Insertion	Station 2: Tip Position	Topic Description: The vitality of PICC insertion confirmation will be identified through Radiography and ECG, with practice of ECG-guided insertion. Topics to be covered: Tip positioning; Radiography; ECG guidance; Guidewire; Saline-Filled Lumen; Tip Movement; Thrombosis; Conclusion.	45 mins	Teaching Method: Clinical station. Learning Outcomes: 1. Knowledge and Understanding: Identify tip confirmation methods. 2. Intellectual Skills: Analyse ECG waveforms. 3. General and Transferable Skills: Apply ECG to confirm PICC tip position.
Day 2: Insertion	Station 3: Securement and Dressing	Topic Description: Proper care, securement, and dressing allow early detection of complications. Topics to be covered: Overview; Purpose of Securement; Types (Gauze, Transparent, Adhesive, Tissue Adhesive, Subcutaneous, Sutures, Add-On Devices); Impact of Inadequate Securement; Dressing Assessment and Changes; Securement Assessment.	45 mins	Teaching Method: Clinical station. Learning Outcomes: 1. Knowledge and Understanding: Identify dressing and securement necessity. 2. Intellectual Skills: Analyse dressing/securement needs by patient condition. 3. General and Transferable Skills: Perform securement and dressing.
Day 2: Insertion	Station 4: Infection Prevention	Topic Description: PICCs must be aseptically cared for and maintained. Infection prevention is crucial. Topics to be covered: Hand Hygiene; Maximal Barrier Precautions (Sterile Gown, Gloves, Drapes); Patient Skin Preparation; Skin Decontamination; Ultrasound; Insertion Checklists; Aseptic Non-Touch Technique (ANTT).	45 mins	Teaching Method: Clinical station. Learning Outcomes: 1. Knowledge and Understanding: Identify infection prevention methods. 2. Intellectual Skills: Analyse disinfection procedure. 3. General and Transferable Skills: Perform sterile insertion with checklists.
Day 3: Post Insertion	Station 5: Complications	Topic Description: Complications during/after insertion and management. Topics to be covered: Insertion-related complications; Post-insertion complications; Any time complications; Summary.	45 mins	Teaching Method: Clinical station. Learning Outcomes: 1. Knowledge and Understanding: Recognise complications. 2. Intellectual Skills: Analyse prevention strategies. 3. General and Transferable Skills: Manage complications.
Day 3: Post Insertion	Station 6: Catheter Function and Device Necessity	Topic Description: When and how to safely remove a PICC; assess function and complications. Topics to be covered: Assessment; Frequency; Insertion site inspection; Tubing/Giving Set Labelling; Occlusions; Extravasation; PPE; Aseptic Technique; Device Necessity; Care Bundles.	45 mins	Teaching Method: Clinical station. Learning Outcomes: 1. Knowledge and Understanding: Recognise function assessment and occlusion types. 2. Intellectual Skills: Decide on device necessity. 3. General and Transferable Skills: Perform safe PICC removal, manage occlusions.
Day 3: Post Insertion	Station 7: Hub Disinfection, Management, and Flushing	Topic Description: Hub disinfection, PICC site assessment, flushing methods. Topics to be covered: Disinfectants; Methods; Changing connectors; Site assessment procedure; Documentation; Flushing rationale; Flushing principles; Volumes; Heparin vs saline; Clearing occlusions.	45 mins	Teaching Method: Clinical station. Learning Outcomes: 1. Knowledge and Understanding: Identify hub disinfection, assessment, and flushing. 2. Intellectual Skills: Correlate site assessment with complication reduction. 3. General and Transferable Skills: Perform flushing and site assessment.
Day 4: Implementation	Role Play Scenarios + Practical	Topic Description: Case scenarios (insertion, complication, post-insertion) and practical implementation of the full PICC process. Topics to be covered: 3 case scenarios; Full insertion, management, removal; Adaptable to group needs.	2 hrs + 3x30 mins	Teaching Method: Clinical station + Role Play. Learning Outcomes: 1. Knowledge and Understanding: Discuss scenarios. 2. Intellectual Skills: Link insertion, management, complication prevention. 3. General and Transferable Skills: Perform insertion/removal across scenarios.
Day 5: Assessment	Assessment 1–3	Topic Description: Theoretical and practical assessments. Topics to be covered: Assessment 1: Theoretical Assessment (30 mins); Assessment 2: Practical Assessment (30 mins); Assessment 3: Logbook Completion (1 month).	30 mins + 30 mins + 1 month	Teaching Method: Written and practical assessments. Learning Outcomes: 1. Knowledge and Understanding: Demonstrate theoretical knowledge. 2. Intellectual Skills: Apply procedural reasoning. 3. General and Transferable Skills: Complete supervised insertions (logbook).

Appendix E: Final PICC three-day curriculum outcome timetables

**Table 10:** Summary of the Final Validated PICC Training Curriculum

Day	Key activities	Time allocation
Day 1	Anatomy, vascular devices, patient selection; ultrasound guidance; tip positioning; securement; infection prevention (with breaks)	7.5 hrs
Day 2	Post-insertion care; complication management; catheter function; hub disinfection; role-play scenarios	7.5 hrs
Day 3	Patient demonstrations; small-group practice; theory and skills assessments; wrap-up (with breaks)	8 hrs
Follow-up	Logbook of ≥5 supervised patient insertions	6 months

Appendix F 

**Table 11:** Detailed Timetable of the Final PICC Curriculum

Day / Module	Topic	Description / Content	Time	Teaching Method / Learning Style / Outcomes
Day 1 – Pre-course Evaluation	Pre-course Survey	Baseline questionnaire to assess participants’ knowledge, skills, confidence, and attitudes toward PICC use before the first lecture.	5 mins	Teaching Method: Structured survey (online or paper). Learning Style: Self-assessment, reflection. Learning Outcomes/Goals: 1. Knowledge and Understanding: Document baseline knowledge. 2. Intellectual Skills: Identify initial confidence/fears. 3. General and Transferable Skills: Promote reflection on learning needs.
Day 1 – Module 1: Assessment & Selection	Lecture I: Vascular Anatomy and Physiology	Topic Description: General understanding of the Anatomy and Physiology of the circulatory system. Topics covered: Vessel Wall Structure; Large veins of the Upper Arm; Veins for PICC; Thoracic vessels; Nerves; Physiology; Virchow’s Triad; Variations by Age Group.	45 mins	Teaching Method: Lecture. Learning Style: Theoretical lecture, visual aids, brainstorming, Q&A. Learning Outcomes/Goals: 1. Knowledge and Understanding: Identify vessel-wall structure and veins. 2. Intellectual Skills: Analyse venous anatomy and physiology in relation to PICC. 3. General and Transferable Skills: Apply vein selection behavior.
Day 1 – Module 1: Assessment & Selection	Lecture II: Vascular Access Devices	Topic Description: Types of vascular devices and choosing the appropriate device. Topics covered: Types of Vascular Access Devices; PICCs; Multi-lumen catheters; Infusate and catheter features; Length of therapy; Pediatric considerations.	45 mins	Teaching Method: Lecture. Learning Style: Theoretical lecture, visual aids, Q&A. Learning Outcomes/Goals: 1. Knowledge and Understanding: Identify vascular devices and selection criteria. 2. Intellectual Skills: Analyze factors in device choice. 3. General and Transferable Skills: Apply decision-making to select devices.
Day 1 – Module 1: Assessment & Selection	Lecture III: PICC Insertion Preparation	Topic Description: Required steps to evaluate patient, obtain consent, perform sterile barrier. Topics covered: Insertion preparation; Informed consent; Pre-insertion assessment; Education; Positioning; Measuring techniques; Sterile barrier precautions.	60 mins	Teaching Method: Lecture. Learning Style: Lecture with diagrams and Q&A. Learning Outcomes/Goals: 1. Knowledge and Understanding: Identify pre-insertion assessments. 2. Intellectual Skills: Analyze patient/device matching and sterilization steps. 3. General and Transferable Skills: Promote sterile environment for PICC insertion.
	Break		15 min	
Day 1 – Module 2: Insertion	Station 1: Ultrasound Guided PICC Insertion	Topic Description: Bedside ultrasound for PICC placement. Topics covered: Basics of ultrasound, vein imaging, probe use, step-by-step modified Seldinger technique.	90 min	Teaching Method: Clinical station. Learning Style: Demonstration + mannequin practice. Learning Outcomes/Goals: 1. Knowledge: Outline ultrasound basics. 2. Intellectual: Analyze step-by-step US-guided insertion. 3. Transferable: Practice probe/needle handling.
Day 1 – Module 2: Insertion	Station 2: Tip Position	Topic Description: Confirm PICC insertion with radiography and ECG. Topics covered: Tip positioning methods, ECG guidance, radiography confirmation, complications.	90 min	Teaching Method: Clinical station. Learning Style: Demonstration + practice. Learning Outcomes/Goals: 1. Knowledge: Identify tip confirmation methods. 2. Intellectual: Analyze ECG/radiography waveforms. 3. Transferable: Apply ECG for tip location.
	Break		15 min	
Day 1 – Module 2: Insertion	Station 3: Securement and Dressing	Topic Description: Proper securement and dressing to prevent complications. Topics covered: Securement types; Transparent dressings; Sutures; Dressing changes.	85 min	Teaching Method: Clinical station. Learning Style: Demonstration + practice. Learning Outcomes/Goals: 1. Knowledge: Identify securement and dressing methods. 2. Intellectual: Analyze dressing choice per patient. 3. Transferable: Perform dressing and securement.
End of Day 1			7.5 hrs	
Day 2 – Module 2: Insertion	Station 4: Infection Prevention	Topic Description: Infection prevention measures during PICC use. Topics covered: Hand hygiene; Sterile gowning; Drapes; Skin preparation; Checklists.	75 mins	Teaching Method: Clinical station. Learning Style: Demonstration + practice. Learning Outcomes/Goals: 1. Knowledge: Identify infection prevention methods. 2. Intellectual: Analyse asepsis protocols. 3. Transferable: Perform sterile insertion with a checklist.
Day 2 – Module 3: Post insertion	Station 5: Complications	Topic Description: Complications during/after insertion and their management. Topics covered: Insertion-related, post-insertion, and late complications.	90 mins	Teaching Method: Clinical station. Learning Style: Demonstration + scenarios. Learning Outcomes/Goals: 1. Knowledge: Recognize complications. 2. Intellectual: Analyze prevention/management strategies. 3. Transferable: Manage complications safely.
Day 2 – Module 3: Post insertion	Station 6: Catheter Function & Device Necessity	Topic Description: Assessing catheter function and determining device necessity. Topics covered: Site assessment; Occlusion types; Device removal criteria.	90 min	Teaching Method: Clinical station. Learning Style: Demonstration + supervised practice. Learning Outcomes/Goals: 1. Knowledge: Recognize catheter function and occlusion. 2. Intellectual: Decide on PICC removal necessity. 3. Transferable: Perform safe PICC removal.
	Break		15 min	
Day 2 – Module 3: Post insertion	Station 7: Hub Disinfection, Management, and Flushing	Topic Description: Hub disinfection protocols and flushing methods. Topics covered: Disinfection methods; Needle-free connectors; Flushing rationale and techniques.	90 min	Teaching Method: Clinical station. Learning Style: Demonstration + practice. Learning Outcomes/Goals: 1. Knowledge: Identify disinfection and flushing procedures. 2. Intellectual: Correlate site assessment with complication prevention. 3. Transferable: Perform hub disinfection and flushing.
Day 2 – Module 4: Implementation	Role Play Scenarios	Topic Description: Clinical scenarios covering insertion, complications, and removal. Topics covered: Scenario 1: Full insertion/management; Scenario 2: Complication during insertion; Scenario 3: Post-insertion complication.	90 min	Teaching Method: Role play. Learning Style: Facilitation and delegation. Learning Outcomes/Goals: 1. Knowledge: Discuss scenarios of PICC use. 2. Intellectual: Link steps of insertion, management, and prevention. 3. Transferable: Perform insertion/removal in simulated cases.
End of Day 2			7.5 hrs	
Day 3 – Documentation, Demonstration, and Assessment	Patient Documentation	Topic Description: Proper documentation of PICC procedures. Topics covered: Documentation of patient/device/insertion/follow-up.	30 mins	Teaching Method: Brainstorming + demonstration. Learning Style: Observation + facilitation. Learning Outcomes/Goals: 1. Knowledge: Identify documentation needs. 2. Intellectual: Analyze importance of documentation. 3. Transferable: Document PICC use accurately.
Day 3 – Documentation, Demonstration, and Assessment	Patient Demonstration	Topic Description: Instructor demonstrates PICC insertion on real patients. Topics covered: Two patient demonstrations (slow-paced and normal-paced).	90 mins	Teaching Method: Demonstration. Learning Style: Observation. Learning Outcomes/Goals: 1. Knowledge: Recognize step-by-step live insertion. 2. Intellectual: Correlate demonstration with learning. 3. Transferable: Observe and apply in practice.
Day 3 – Documentation, Demonstration, and Assessment	Practice Time	Topic Description: Hands-on practice in pairs with mannequins under supervision.	3 hrs	Teaching Method: Skill lab. Learning Style: Simulation practice. Learning Outcomes/Goals: 1. Knowledge: Apply learned skills. 2. Intellectual: Consolidate technique. 3. Transferable: Perform supervised practice.
	Break		50 min	
Day 3 – Documentation, Demonstration, and Assessment	Summative Assessment	Topic Description: Theoretical and OSCE-based assessments. Topics covered: Written exam, mannequin insertion OSCE, feedback session, logbook instructions.	2 hrs	Teaching Method: Examination + feedback. Learning Style: Written + practical OSCE. Learning Outcomes/Goals: 1. Knowledge: Demonstrate theoretical understanding. 2. Intellectual: Show procedural reasoning. 3. Transferable: Perform supervised insertions with competence.
Day 3 – Post-course Evaluation	Post-course Survey & Feedback	Post-training questionnaire to reassess knowledge, skills, and attitudes; structured participant feedback on course content, teaching methods, and logistics.	10 min	Teaching Method: Structured survey + feedback forms. Learning Style: Reflective evaluation. Learning Outcomes/Goals: 1. Knowledge and Understanding: Identify learning gains. 2. Intellectual Skills: Reflect on skill acquisition. 3. General and Transferable Skills: Provide constructive feedback for course improvement.
End of Day 3			8 hrs	

Appendix G 

**Table 12:** Assessment and Certification Criteria for the Final PICC Curriculum

Assessment component	Requirement	Passing criteria	Time allocation	Notes
Attendance	Participation in all course sessions	≥75% attendance required	—	Absence beyond 25% results in ineligibility for certification
Theoretical exam	Written multiple-choice/short-answer exam (50 MCQs)	≥70% pass mark	1 hr	Covers anatomy, indications, complications, and infection prevention
Simulation assessment	Mannequin-based insertion practice	Minimum of 5 mannequin insertions (up to 2 reattempts allowed)	Across Day 1 and 2	Supervision by certified instructors with structured OSCE-style checklists
Practical OSCE exam	Structured observed practical exam (mannequin insertion + skills checklist)	Demonstration of procedural competence	1 hr	Conducted during the Day 3 summative assessment
Clinical logbook	Supervised patient insertions	≥5 successful supervised insertions over 6 months	6 months	Validated by certified supervisors; feedback documented in logbook
Professional skills	Communication, teamwork, adherence to sterile technique	Competent demonstration required	Observed throughout the course	Assessed informally during role-play, simulation, and clinical practice
